# Rapid Weight Changes and Competitive Outcomes in Muay Thai and Mixed Martial Arts: A 14-Month Study of 24 Combat Sports Events

**DOI:** 10.3390/sports12100280

**Published:** 2024-10-16

**Authors:** Colin S. Doherty, Lauren V. Fortington, Oliver R. Barley

**Affiliations:** School of Medical and Health Sciences, Edith Cowan University, Joondalup, WA 6027, Australia; l.fortington@ecu.edu.au (L.V.F.); o.barley@ecu.edu.au (O.R.B.)

**Keywords:** rapid weight loss, weight cutting, rapid weight gain, performance

## Abstract

This study investigates the rapid weight loss (RWL) and rapid weight gain (RWG) of contest winners and losers from Muay Thai (MT) and mixed martial arts (MMA) events. The relationship between rapid weight change variables of males and females, and competitive success is also examined. Data from a weight management questionnaire was collected one day post-competition from 185 participants across 24 fight events, resulting in 263 responses (MMA: n = 78, MT: n = 185). Official and secondary weigh-in data were provided by the Combat Sports Commission. The results show that in MT, contest winners compared to losers had significantly greater RWL–7 days (5.9 ± 2.6% vs. 4.6 ± 2.7%, *p* = 0.01), RWG (6.2 ± 2.9% vs. 4.7 ± 2.8%, *p* = 0.003), and RWG/RWL ratio (108% [86–132%] vs. 86% [60–119%], *p* = 0.038), while no significant differences were observed for MMA. Mixed logistic regression models that controlled for age showed that a 1% increase in female RWL–24 h ([OR = 1.57, R^2^ = 0.105, *p* = 0.001]) was associated with a 1.6 times higher likelihood of winning compared to female athletes without this increase. We emphasise that associations do not imply causation, and it is possible that other factors which align with rapid weight change practices may impact the observed relationship. Nonetheless, MT contest winners show greater RWL, RWG, and RWG/RWL ratio than losers, and female rapid weight changes appear to be associated with competitive success in this cohort.

## 1. Introduction

Combat sports competitors are commonly matched based on key characteristics, including body mass (BM), sex, and fight experience. Prior to each contest, official weigh-ins are conducted to check whether the athlete’s BM complies with their chosen weight class. The timing of these weigh-ins can vary, taking place either minutes before competition or up to 32 h prior [1,2], depending on factors such as the combat sport, commission legislation, and promotion preferences [3]. In preparation for the weigh-in, many combat athletes employ rapid weight loss (RWL) strategies [4], such as food and fluid restriction, fasting, heat exposure, and increased exercise [1,2,5]. These methods temporarily reduce BM and aid athletes in meeting their self-selected weight class requirements. Subsequently, after the weigh-in and before competition, athletes aim to replenish fluid and glycogen losses, which results in rapid weight gain (RWG) [2,6,7,8]. Despite potential health risks and performance decrements associated with RWL [1], many athletes believe that RWL followed by RWG offers a competitive advantage [9,10]. To monitor RWG, many combat sports commissions conduct secondary weigh-ins on the day of competition [2,6,7,8,11,12,13]. These secondary weigh-ins have revealed instances where competitors exceeded their official weigh-in BM, sometimes by as much as three weight classes [2,11]. However, it remains unclear whether these rapid and often drastic changes in BM are associated with a competitive advantage across all combat sports and in both males and females.

There is extensive documentation on the negative impact of RWL on performance outcomes in controlled laboratory settings [1,3,14,15,16,17]. Some research suggests that physical and cognitive function can be recovered following rehydration and refuelling, while others still report reduced physical performance [1,16]. A more ecologically valid area of emerging research involves comparing real-world RWL and/or RWG between contest winners and losers [11,12,13,18,19,20,21,22]. For example, in mixed martial arts (MMA), losing athletes tend to undergo a significantly greater magnitude of RWL, averaging 10.6%, compared to winning athletes, who typically reduce their BM by 8.6% [18]. However, other studies have shown no difference or even that winners have greater RWL, highlighting the inconsistencies in findings to date [3]. Alternatively, researchers have also investigated whether RWL is associated with a higher likelihood of winning a contest. A study on MMA athletes reported that for every 1% increase in RWL, the odds of winning decreased by 11% [18]. Other research across various combat sports, including boxing, MMA, and taekwondo, has shown no relationship between RWL and competitive success [19,22,23]. Similarly, investigations into RWG have yielded mixed results. In MMA, greater RWG is associated with winning for regional and international male competitors; however, this association does not extend to female competitors [24]. Conversely, other MMA research shows no significant association between sex, competition level, or BM division and competitive success, but does report an independent effect for RWG and increased probability of winning [13]. Other studies demonstrate benefits in sports such as MMA, judo, and wrestling [13,19,20,24,25], while others report no significant findings in disciplines such as boxing, MMA, and wrestling [11,12,21,26]. To date, the majority of the literature has focused on RWG in MMA and boxing, thus leaving RWL and other combat sports, such as Muay Thai (MT), relatively understudied. This discrepancy may be attributed to the challenges and complexities associated with collecting RWL data [2] in contrast to the accessibility of retrospective RWG data from athletic commissions.

One limitation of the existing literature on rapid weight changes and competitive outcomes is the statistical analysis interpretation and model reporting. For instance, a recent study conducted on a large sample of MMA contests discovered that with every 1% increase in RWG, there was a corresponding 4.5% increase in the probability of winning after controlling for variables such as sex, weight division, and competition level [13]. However, it is important to clarify that the odds ratio (OR) of 1.045 in this study was mistakenly interpreted as a probability. Logistic regression output represents log (odds), which can be transformed into ORs indicating the rate of change in the predictor compared to the base level in odds, rather than directly representing probabilities. Furthermore, other studies [13,18,19,24] lack crucial information regarding the selection and configuration of logistic regression models, their diagnostics, or their level of fit with the data. This lack of essential details makes interpretation challenging and leaves certain characteristics of the models ambiguous. Despite the existence of several investigations examining the effects of RWL on health and performance outcomes [1,3,27,28,29,30,31,32,33,34], no study has explored the relationship between RWL and RWG in MT athletes. Moreover, previous research has indicated a lack of sufficient evidence to use RWG as a proxy for RWL, and there has been an inadequate examination of female athletes [2]. These findings underscore the importance of collecting data on both RWL and RWG as well as the need to study female athletes.

To overcome some of the limitations found in the existing literature, this study aimed to investigate RWL–7 days and RWL–24 h prior to official weigh-ins, RWG between official and secondary weigh-ins, and the RWG/RWL ratio among winners and losers in both MMA and MT. Furthermore, the study explored the potential association between these variables and the likelihood of winning a contest for male and female subgroups separately. It was hypothesized that there would be no discernible differences in rapid weight changes between contest winners and losers, and that these variables would not significantly explain contest outcomes. By conducting this investigation, we aimed to provide valuable insights to coaches, athletes, and regulatory bodies regarding the typical magnitudes of rapid weight changes and their associations with competitive success.

## 2. Methods

### 2.1. Study Design and Ethics

This 14-month observational study focused on MMA and MT competitors across 24 combat sports events in Western Australia. The study received approval from Edith Cowan University’s Human Research Ethics Committee (Research Ethics Identification: 2022-03382-DOHERTY). Digital informed consent was obtained from all participants, and a data-sharing agreement was in place between ECU and the Western Australia Combat Sports Commission (WACSC)

### 2.2. Recruitment and Participants

The principal investigator (CD) utilised various communication channels, including social media, phone, and email, to reach out to coaches and event promoters to inform them about the study and request assistance in recruiting participants. Inclusion criteria required participants to have competed in a sanctioned MMA or MT contest regulated by the WACSC, be at least 18 years old, and have a sufficient understanding of English to provide informed consent and complete a questionnaire. It is important to note that the WACSC does not distinguish between competitive levels (amateur and professional) due to their legislative requirements, which prioritise safety, health, and integrity equally across all levels of competition. Consequently, the data are presented as a single group comprising competitors from regional, national, and international levels. However, it is worth mentioning that data were not available for every contest winner and loser from each bout. Therefore, although it would have been the preferred approach, weight differentials between contestants could not be calculated [24].

### 2.3. Reporting of Body Mass

Participants were sent a weight management questionnaire (WMQ) on the day of the weigh-in or the contest, with the median response time being 1 day (−1, 3 days) post-competition. The WMQ asked athletes what their BM was at 7 days and 24 h before the weigh-in as well as when the notice was received (51% received 6–8 weeks) for the contest (Supplementary File S1). Participants were not provided with specific instructions on how to collect their BM; however, because MMA and MT are weight-category sports, these athletes typically conduct BM checks under similar conditions to the official weigh-in. The WACSC supplied the official weigh-in BM, secondary weigh-in BM, and fight finish details. Official weigh-ins were conducted by a WACSC appointee, with all competitors required to undergo both an initial (official) weigh-in and a pre-competition (secondary) weigh-in. The official weigh-in took place no earlier than 24 h before the advertised start time of the contest. Male contestants were required to wear light underwear or similar attire, while female contestants wore light underwear and a bra top or similar attire, with no shoes or socks permitted. Each participant was allowed only one attempt at the weigh-in and was given a 500 g BM allowance. A secondary weigh-in was conducted to determine the BM regained since the official weigh-in. The secondary weigh-in occurred no earlier than the advertised start time of the event (doors open) and no later than 2 h after the advertised start time. Unlike the initial weigh-in, participants were not required to meet specific BM requirements at the secondary weigh-in. In all instances, the official weigh-ins were conducted the day before the event. The average time difference between the official and secondary weigh-ins was 21.5 ± 2.3 h (minimum: 14.6 h, maximum: 26.5 h). Both the initial and secondary weigh-ins used an official event digital scale (Wedderburn, WM206, Tanita, Perth, Australia), calibrated every six months to ensure accuracy and consistency.

### 2.4. Data Handling and Definitions

In this study, RWL is defined as any change in BM occurring in the 7 days preceding the official weigh-in, while RWG refers to any change in BM from the official weigh-in up until the contest [2]. The formulas used for calculating RWL–7 days and RWL–24 h are as follows [2]:

$$RWL-7\;days\%=\left(\frac{BM-7\;days\;minus\;Official\;weigh–in\;BM}{Official\;weigh–in\;BM}\right)\times 100$$

$$RWL-24\;hours\%=\left(\frac{BM-24\;h\;minus\;Official\;weigh–in\;BM}{Official\;weigh–in\;BM}\right)\times 100$$

Similarly, RWG was determined using

$$RWG\%=\left(\frac{\left(Secondary\;weigh-in\;BM\;minus\;Official\;weigh–in\;BM\right)}{Official\;weigh–in\;BM}\right)\times 100$$

Subsequently, the RWG/RWL ratio was calculated by dividing the RWG by the RWL–7 days, which provides insight into how much of the lost BM was regained before competition. To our knowledge, we are the first study to investigate the RWG/RWL ratio.

**Figure 1:**
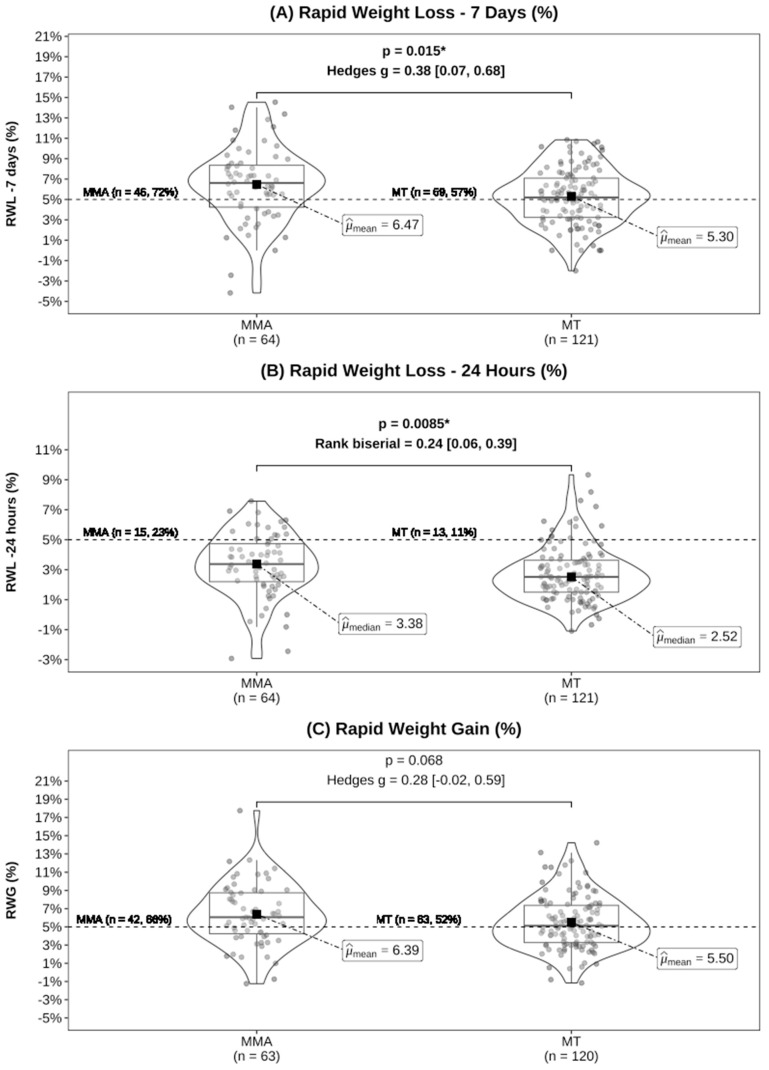
(**A**) Rapid weight loss–7 days (RWL–7 days), (**B**) rapid weight loss–24 h (RWL–24 h), and (**C**) rapid weight gain (RWG) comparisons between mixed martial arts (MMA) and Muay Thai (MT). Effect sizes were calculated using Hedges’ g for mean comparisons and rank biserial for median comparisons [95% CIs]. The dashed line and labels show the count and proportion of athletes at or above 5%, and * indicates *p* < 0.05.

Consistent with previous research, we calculated the count and proportions of athletes experiencing changes of 5% or more in these variables (Figure 1) [24]. Notably, heavyweight athletes have typically been excluded from similar analyses in previous research [13,24]. We chose to include the heaviest athletes from our analysis because they also engaged in RWL and RWG practices. To accurately reflect the real-world practices of the studied athletes, all observations with negative and 0% rapid weight change variables were included. However, in our dataset, which includes a combination of MMA and MT data with different weight classes, BM categories (Light, Medium, and Heavy) were assigned based on the official weigh-in BM, resulting in the following ranges: Light (45.5–66.8 kg), Medium (67.4–87.4 kg), and Heavy (92.4–110 kg). Data were categorised using the cut function in R.

### 2.5. Data Analysis

Descriptive analyses were conducted for both MMA and MT contest winners and losers. The normality and homogeneity were assessed using the Shapiro–Wilk and Levene’s test, respectively. Normally distributed variables are presented as the means ± standard deviations with Hedges’ g effect sizes and were analysed using two-sample *t*-tests. Non-normally distributed variables are reported as the medians (interquartile ranges) with rank biserial effect sizes and were examined with the Wilcoxon rank sum test. However, due to the limited number of female MMA athletes (n = 5), statistical comparisons between contest winners and losers were not conducted for this subgroup. All box–violin plots were created using the ‘ggstatsplot’ package in the R statistical language program (version 4.3.2; R Core Team, 2023) [35].

To assess the association between rapid weight change variables and the likelihood of winning a contest, we used four separate mixed-effects logistic regression models. Sex-specific analyses were conducted due to prior research highlighting different findings for males and females [13,24]. Age was controlled for as previous research indicates it can differ between contest winners and losers [12]. However, further stratification by sport (MMA or MT) was not performed due to the limited number of female MMA athletes. This approach enabled a larger sample size and increased the statistical power to detect independent associations, if they existed, between male and female rapid weight changes and competitive success. Mixed models were selected for their ability to consider both fixed effects (rapid weight changes by sex and age) and random effects (participant), which is especially important when dealing with repeated observations. Specifically, four models were configured, each controlling for age and separate effects for males and females: (1) RWL–7 days, (2) RWL–24 h, (3) RWG, and (4) the RWG/RWL ratio. It is important to note that our models were designed to assess overall associations rather than make predictions or develop predictive models, which are best suited to larger sample sizes. To select the final models, we used the ‘compare_performance’ function and performed diagnostic checks using the ‘check_model’ function from the ‘performance’ package. Model diagnostics included visualising posterior predictors, binned residuals, influential observations, collinearity, normality of residuals, and random effects (Supplementary File S2). The results are presented as ORs with 95% confidence intervals (CIs). The regression table was created using the ‘tab_model’ function from sjPlot, and the marginal (fixed effects) and conditional (fixed and random effects) pseudo coefficients of determination (pseudo R^2^) were calculated based on Nakagawa et al. [36]. Statistical significance was set a priori at *p* < 0.05. All statistical analyses and visualisations were carried out using the R statistical language program.

## 3. Results

We collected a total of 295 responses from the WMQ over 14 months. Only MMA and MT were studied, as the sample sizes for boxing and kickboxing were small. After excluding 21 responses from boxing, 1 from kickboxing, 7 draws, and 3 individuals under 18 years old, 263 data points remained for analyses. After removing repeat observations, group and subgroup comparisons were conducted on a sample of 185 athletes (Table 1). The full dataset was only used (n = 263) for mixed logistic regression analyses, and contained 204 males (winners = 106, losers = 98) and 59 females (winners = 36, losers = 23).

Specifically, 72% of MMA athletes and 57% of MT athletes displayed an RWL–7 days greater than 5%. Similarly, 23% of MMA athletes and 11% of MT athletes had an RWL–24 h greater than 5%. Regarding RWG, 66% of MMA athletes and 52% of MT athletes showed an RWG greater than 5%. On average, MMA athletes displayed approximately 1.2% greater RWL–7 days and 0.9% greater RWG compared to MT athletes (Figure 1). However, two athletes did not show up to the secondary weigh-ins, so their RWG could not be calculated. Additionally, five athletes had an RWL of 0%, so their RWG/RWL ratio could not be calculated (Table 2).

### 3.1. Group and Subgroup Comparisons

**Figure 2:**
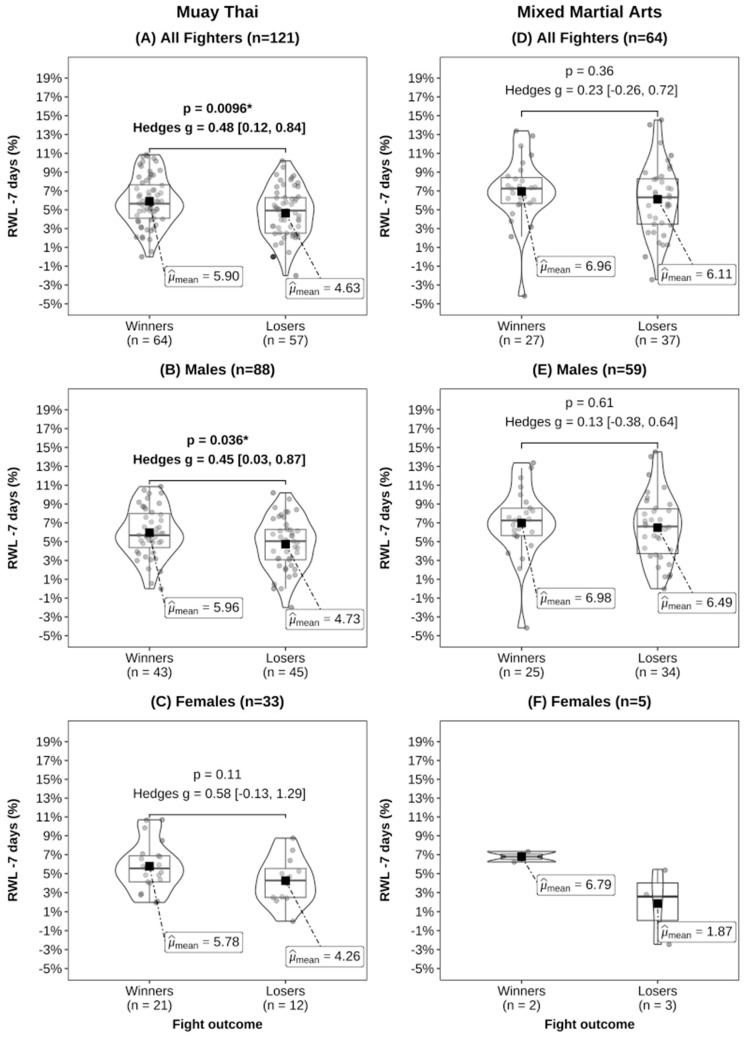
Rapid weight loss–7 days (RWL–7 days) by fight outcome for Muay Thai (**A**–**C**) and mixed martial arts (**D**–**F**). Effect sizes were calculated using Hedges’ g for mean comparisons [95% CIs]. Statistical comparisons were not performed for females in the MMA group due to the small sample size , and * indicates *p* < 0.05.

**Figure 3:**
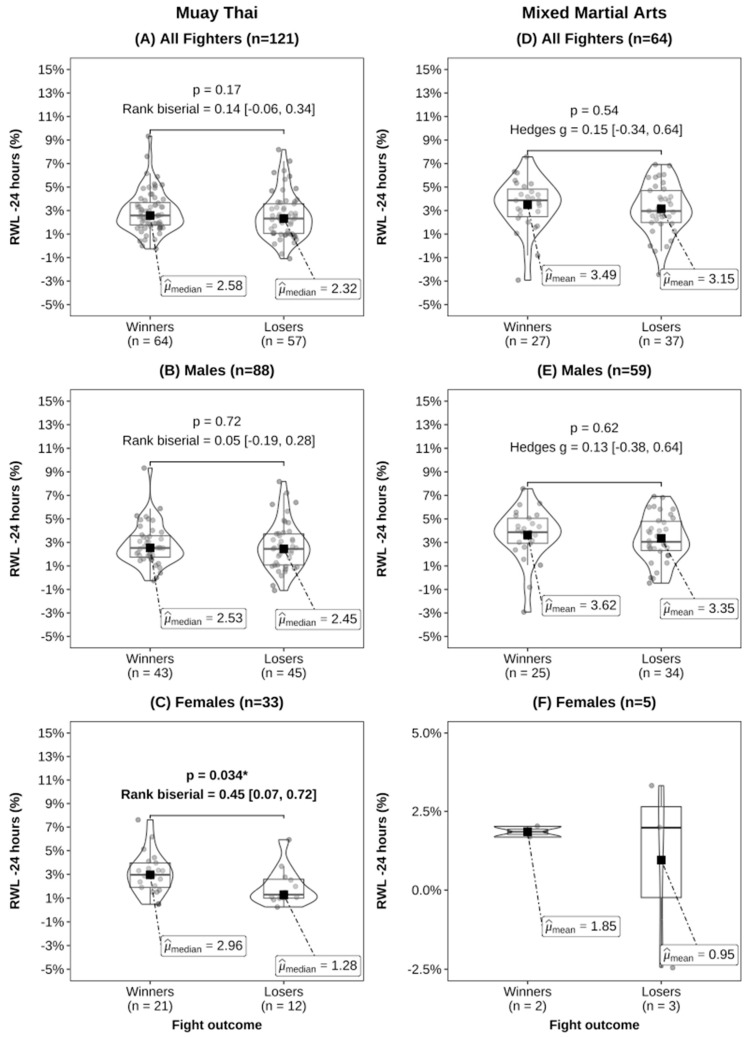
Rapid weight loss–24 h (RWL–24 h) by fight outcome for Muay Thai (**A**–**C**) and mixed martial arts (**D**–**F**). Effect sizes were calculated using Hedges’ g for mean comparisons and rank biserial for median comparisons [95% CIs]. Statistical comparisons were not performed for females in the MMA group due to the small sample size , and * indicates *p* < 0.05.

**Figure 4:**
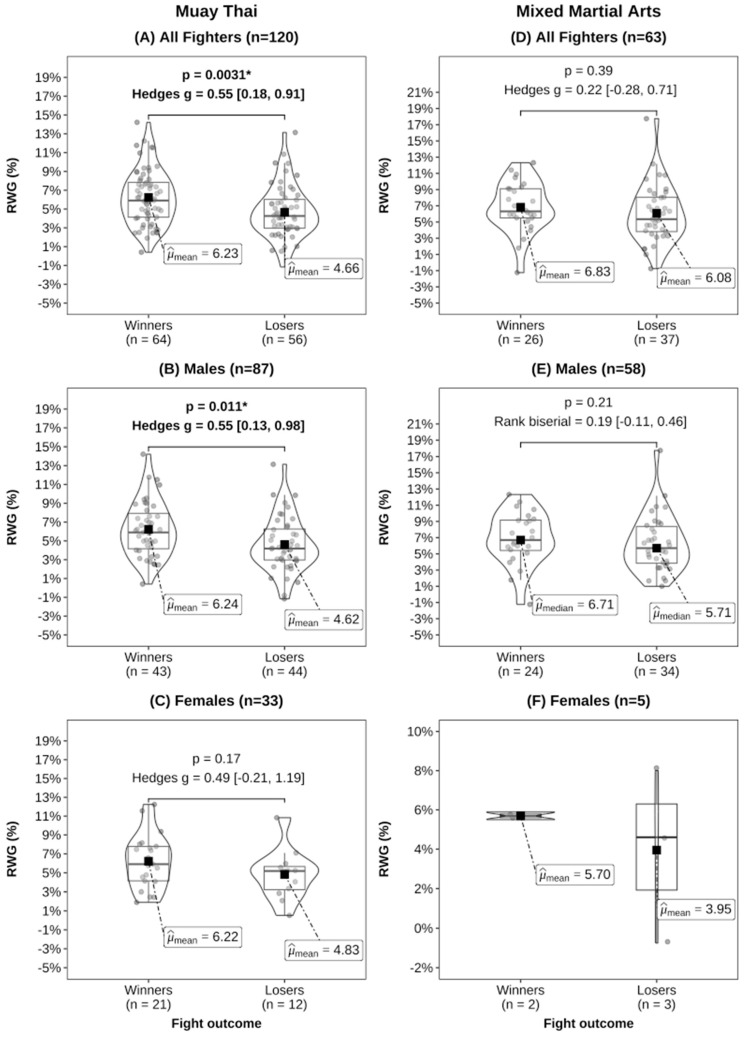
Rapid weight gain (RWG) by fight outcome for Muay Thai (**A**–**C**) and mixed martial arts (**D**–**F**). Effect sizes were calculated using Hedges’ g for mean comparisons and rank biserial for median comparisons [95% CIs]. Statistical comparisons were not performed for females in the MMA group due to the small sample size, and * indicates *p* < 0.05.

In our analysis comparing contest winners with losers in both MMA and MT, no significant differences were observed in absolute BM at any time point (Table 2). However, winners in MT exhibited significantly greater RWL–7 days compared to losers (Figure 2). Additionally, for RWL–24 h, only female contest winners in MT displayed higher values than losers (Figure 3). Moreover, we found that contest winners in MT, as well as male MT athletes, exhibited significantly greater RWG compared to losers (Figure 4). No statistically significant differences were detected in MMA athletes or male and female subgroups.

### 3.2. Logistic Regression Analyses

Logistic regression analyses were conducted with slightly different sample sizes due to missing values. Models 1 and 2 used the full sample (263 observations). Model 3, had two missing RWG values, and was based on 261 observations. Similarly, Model 4 excluded eight responses for the RWG/RWL ratio (seven due to 0% RWL and one influential observation), resulting in a sample size of 253 observations. It is important to note that model accuracy may be less precise at the lower and higher extremes of rapid weight change values (Supplementary File S2). However, the primary purpose of the models was to examine overall associations rather than make precise predictions. Notably, all female rapid weight change variables, when controlled for age, were significantly associated with winning a contest, whereas this association was not observed for males. Additionally, age exhibited a significant effect on all variables, indicating that younger athletes had higher odds of winning (Table 3). However, the variance in the fight outcome explained by the fixed effects (marginal R^2^) was low, ranging from 7% to 11%.

## 4. Discussion

The objective of this study was to compare RWL–7 days, RWL–24 h, RWG, and the RWG/RWL ratio among winners and losers in MMA and MT contests. Additionally, we investigated the relationship between these variables and the likelihood of winning a contest separately for males and females. Our analyses revealed that in MT, but not in MMA, contest winners exhibited significantly higher values for RWL–7 days and RWG and greater RWG/RWL ratio compared to losers. Similarly, the MT subgroup analyses showed that male contest winners had greater levels of RWL–7 days and RWG, while female contest winners had greater RWL–24 h and RWG compared to losers. However, after controlling for age, the mixed logistic regression models showed that only the rapid weight change variables in females were significantly associated with winning a contest. The greatest effect was observed for female RWL–24 h, where a 1% increase was associated with a 1.6 times higher likelihood of winning a contest (OR = 1.57, R² = 0.105, *p* = 0.001) compared to those without this increase. It is important to mention that these findings should be replicated in future studies, as our female sample size was relatively small (38 athletes, 59 observations, 5 from MMA). It is important to emphasise that while rapid weight changes in females are linked to competitive success, it remains unclear whether other unexplored factors may influence and potentially distort the observed relationships.

### 4.1. Group and Subgroup Comparisons

When comparing rapid weight changes by sport, our findings show that MMA athletes had a 1.2% greater RWL–7 days and a 0.86% greater RWL–24 h compared to MT athletes. However, this difference was not observed for RWG. These findings align with previous research that suggests MMA athletes generally undergo more substantial RWL in comparison to MT athletes [4]. This observation may be attributed to MMA competitors having an out-of-competition BM differential of 11.3 kg compared to their current weight class, while in MT, this differential is only 4.6 kg. [4]. Nevertheless, despite the greater rapid weight changes in MMA athletes, we only found significant differences between contest winners and losers in MT. Specifically, the RWG/RWL ratio was 22% greater for MT winners compared to losers, whereas in MMA it was only 3% higher for winners and non-significant. This suggests that MT winners tend to fully recover from or even surpass their levels of RWL–7 days, whereas losers do not. Therefore, MT contest winners may implement more effective rehydration and refuelling practices than losers which could provide a performance advantage, a finding that is supported by previous research [19]. To the best of our knowledge, the present study is the first to examine the RWG/RWL ratio. However, noteworthy observations were made during the calculation of this ratio for previous research. Brechney et al. [18] reported that RWL was higher for contest losers (10.6%) compared to winners (8.6%). However, when computing the RWG/RWL ratio, winners had a ratio of 79% (6.8% RWG/8.6% RWL) while losers had a ratio of 70% (7.4% RWG/10.6% RWL), indicating a slightly higher ratio for winners [18]. Similarly, in a study conducted by Coswig et al. [19], the RWG/RWL ratio for winners was 90% (6.4% RWG/7.1% RWL), whereas for losers it was 34% (2.6% RWG/7.7% RWL). These findings suggest that a higher, and sometimes much higher, RWG/RWL ratio is characteristic of contest winners, potentially attributable to superior post-weigh-in recovery practices [19].

The subgroup analyses for male and female athletes in MMA and MT revealed interesting differences in rapid weight changes. In MMA, no differences were found. However, compared to contest losers in MT, female winners had a 1.7% greater RWL–24 h, while male winners had a 1.2% and 1.6% higher RWL–7 days and RWG. Female contest winners also had a 1.4% greater RWG compared to losers, but this did not reach statistical significance. These RWG findings suggest that both male and female MT contest winners may have achieved better rehydration and muscle glycogen recovery than the losers. This is in line with research in MMA, which found that contest winners had higher total energy and carbohydrate intake during the RWL and RWG phase, and greater RWG was associated with increased high-intensity efforts and lower limb combinations [19], which are also important for MT performance. Therefore, MT contest winners may be implementing superior recovery practices after weigh-ins, potentially providing a performance advantage. Alternatively, it may be the case that contest losers do not recover as well as winners between weigh-ins and competition, which may lead to impaired performance. However, sex-specific RWL and RWG strategies beyond the prevalence and magnitude need to be studied in future research. Nonetheless, we believe that the RWG/RWL ratio is likely the most informative variable for capturing the effect of rapid weight changes on competitive outcomes. Examining RWL without considering RWG, or vice versa, only provides one dimension of the BM changes pre-competition. Therefore, it is crucial for future research to collect data on both RWL and RWG and examine the RWG/RWL ratio across diverse cohorts. Additionally, research reporting energy, fluid and nutrient consumption is needed to provide insight and context to rapid weight change data reported in combat sports. Such an approach will enable a more comprehensive understanding of the intricate relationship between rapid weight changes and competitive success.

The notice provided to athletes by the promoter prior to a fight has the potential to impact RWL practices and, subsequently, the athletes’ ability to recover before the competition. Our findings indicate that 29% of athletes received a notice period of 0–5 weeks, while the majority were given more than 5 weeks’ notice. Previous studies have suggested that MMA athletes experience a BM increase of 7.8 kg within 7 days after a competition, as opposed to only 4.6 kg in the case of MT athletes [4]. Taking into account this rebound in BM, athletes with shorter notice periods and closer intervals between competitions may need to adopt more aggressive RWL strategies in comparison to those who have more than 5 weeks’ notice. Future research should examine whether the duration of fight notice and the initial BM at the start of fight camp influence the gradual and rapid weight change strategies employed by athletes and, ultimately, their relationship with competitive success.

### 4.2. Logistic Regression Analyses

In the logistic regression analyses conducted on the entire sample size (n = 263), we found that the variance of the fixed effects in the models, which explain fight outcomes, was relatively low, ranging from 10% to 11%. Hence, it is important to exercise caution when interpreting these results. Notably, after controlling for age, we observed significant associations between female RWL variables and the odds of winning a contest. However, in practical terms, a mere 1% variance in RWL is unlikely to have a meaningful impact on fight outcomes. It is plausible that factors such as fight experience, skill level, and other relevant variables may be intertwined with RWL, potentially confounding some of the observed effects. For instance, our analysis revealed a significant independent effect of age, indicating that with each year’s increase in age, the odds of winning decreased by 8–9%. This aligns with findings in professional boxing and MMA, where winners tend to be younger than losers [12,37]. Furthermore, previous investigations have identified determinants of winning outcomes in MMA, such as ground strikes, takedown accuracy, strikes landed, and strike accuracy, which are not accounted for in our models [38]. Therefore, although female RWL may contribute to higher odds of success, it may be associated with other influential attributes that could distort the true relationship between rapid weight changes and contest success. Additionally, in the context of RWL, often overlooked but potentially important differences in sweat rates among females, both in general and possibly varying by menstrual stage, may merit consideration [31,39]. For example, although speculative, contest losers may have slower sweat rates, leading to prolonged heat exposure and/or exercise to achieve comparable sweat volumes to those with faster rates. This could result in greater accumulation of fatigue, posing a substantial obstacle to complete recovery before competition and potentially impairing performance. Consequently, it is recommended that future research explores the intricate interplay between exposure time to heat and/or exercise, athletes’ sweat rates, strategies for recovery, and perceived levels of fatigue prior to competition.

Female RWG and the female RWG/RWL ratio had significant associations with winning a contest, where a 1% increase was found to be associated with 1.3 and 1.01 greater odds of winning a contest. It is important to note, however, that there was a substantial difference in the scale of the RWG/RWL ratio, ranging from −92% to 320%, compared to the narrower range of RWG (−1.2% to 17.8%). Despite both RWG and the RWG/RWL ratio having a unit increase equivalent to 1%, their effects are not proportional due to the variables’ varied ranges. Our findings suggest that the female RWG/RWL ratio is a significant predictor of competitive outcomes, emphasising the importance of BM regained following RWL. Comparing our findings with previous research reveals both similarities and differences. For example, Baribeau et al. [24] reported a significant association between RWG and increased odds of winning for male regional MMA athletes but not for females. In contrast, Kirk et al. [11] found no significant link between RWG and contest outcomes in professional MMA athletes. The conflicting findings between previous research and our study may be attributed to variations in competitive levels [13,24], statistical methodologies, sample sizes, and the inclusion of MT athletes in our sample. It is also important to consider how statistical analyses were performed. Notably, the data from the Baribeau et al. study encompassed observations from 2015 to 2019, potentially including repeat observations from athletes participating in multiple contests [24]. However, the use of standard logistic regression in the Baribeau et al. analysis may lead to biased estimates and incorrect standard errors if correlations exist among observations within the same athlete. In contrast, our study employed a more appropriate approach for data with repeat observations, such as mixed logistic regression, which was also utilised by Faro et al., who found a 4.5% increase in the probability of winning for every 1% of additional RWG, irrespective of competition level, sex, and BM division [13]. Currently, detailed model selection, configuration, and diagnostics are generally lacking in studies investigating competitive outcomes. Future investigations should provide thorough model diagnostics and performance metrics to enhance confidence in the reported findings.

### 4.3. Limitations

While this research possesses notable strengths, it is important to acknowledge several limitations. Firstly, all data on RWL were self-reported. Although there is no reason to suspect deliberate misreporting by athletes, there remains a possibility that some competitors may have inaccurately recalled their BM. Participants were not provided with specific instructions on how to record and report their BM, but due to the nature of weight-category sports, athletes typically follow similar conditions to officials weigh-ins. Secondly, the study’s sample size for female MMA competitors was small, which hindered the ability to make comparisons within this subgroup. Despite our models demonstrating a significant effect for females, the relatively small sample size and the limited explanatory power of the models emphasize the need for further investigation. Furthermore, specific data on the competition level and fight records or experience were not recorded or provided, which is another limitation. Although the athletes participated in a variety of regional, national, and international contests, the absence of detailed information regarding the level of competition and fight experience may impact the generalizability of our findings. It was also unknown how many fights the athletes participated in each year and when their previous contest was held, which has the potential to impact rapid weight change data. Moreover, due to the unavailability of data on all contest winners and losers, we relied on a random sample from 24 fight events. As a result, we were unable to compare weight differentials between contests. Power analyses were not conducted as the number of contests or the level of interest in the study could not be predicted, and post hoc power analyses were not performed as they were inappropriate [40]. Due to the absence of a pre-study power analysis, the research may have been underpowered for some analyses, which poses a risk of failing to detect genuine effects (false negatives) attributable to a limited sample size. Consequently, future research should aim to conduct power analyses and study larger sample sizes. In addition, future research should seek to explore whether specific magnitudes of RWL offer a competitive advantage and investigate the potential consequences of having a BM above or below this range. Further investigation is warranted, particularly for MT athletes, to gain a better understanding of the observed distinctions between female winners and losers.

### 4.4. Practical Applications

Our research indicates notable disparities in rapid weight changes between winners and losers in the sport of MT, but not in the MMA cohort (Figure 2A, Figure 3A and Figure 4A). This suggests that rapid weight change practices may be more consistent among MMA athletes, while greater variability exists within MT. When one athlete achieves incomplete recovery while the other achieves full or greater recovery, the better-recovered athlete may possess a performance advantage, especially in bouts that go the full scheduled distance. For example, we found that MT contest winners replenished 108% of the BM lost in the 7 days before weigh-ins, whereas losers only recovered 86%. However, it is uncommon for athletes to know an opponent’s RWL, RWG, and how recovered they are, thus our recommendation is to compete in a weight class where the athlete feels and performs at their best. They should focus on low-risk strategies for BM loss in the final week before weigh-ins, such as reducing sodium, fibre, and carbohydrate intake while minimising dehydration in the last 24 h [31,41,42]. Athletes and coaches should prioritise evidence-based strategies for rehydration and refuelling to optimize muscle glycogen restoration and hydration status, which are critical for competition performance [31,41,42], and consider consulting with qualified combat sports nutritionists or dieticians to minimise health and performance risks.

## 5. Conclusions

Our findings demonstrate that, on average, MMA athletes have greater RWL in comparison to MT athletes, but there is no significant disparity in RWG between the two groups. In the context of MT, winners of contests displayed significantly greater RWL, RWG, and RWG/RWL ratio than the losers. Specifically, the greater RWG/RWL ratio in MT contest winners may suggest the utilisation of more effective rehydration and refuelling strategies following the weigh-in process. Employing mixed logistic regression models, we also identified that greater RWL, RWG, and RWG/RWL ratio in female participants were associated with higher odds of winning a contest. However, the relatively limited sample size necessitates the replication of these findings. While these associations do not establish a causal relationship, they do highlight characteristics of contest winners that could potentially align with other competitive advantages. Therefore, further investigation is required to explore potential confounding factors. Athletes and coaches should adopt evidence-based practices for RWL and RWG to prioritize athlete health and optimize performance. Adhering to scientifically supported methodologies can mitigate health risks while enhancing the likelihood that athletes are adequately fuelled for competition.

## Figures and Tables

**Table 1:** Overview of participant characteristics and fight details by combat sport and fight outcome. Data are presented as medians (interquartile ranges) and n (%).

	MMA				MT		
Variable	Winners	Losers	Overall		Winners	Losers	Overall
	n = 27	n = 37	n = 64		n = 64	n = 57	n = 121
**Age, years**	25	25	25		26	28	27
	(23, 28)	(22, 32)	(22, 30)		(23, 30)	(22, 32)	(22, 31)
**Sex**							
Male	25 (93%)	34 (92%)	59 (92%)		43 (67%)	45 (79%)	88 (73%)
Female	2 (7%)	3 (8%)	5 (8%)		21 (33%)	12 (21%)	33 (27%)
**Body mass category**							
Light (45.5–66.8 kg)	14 (52%)	17 (46%)	31 (48%)		37 (58%)	34 (60%)	71 (59%)
Medium (67.4–87.4 kg)	12 (44%)	19 (51%)	31 (48%)		24 (38%)	20 (35%)	44 (36%)
Heavy (92.4–110.0 kg)	1 (4%)	1 (3%)	2 (3%)		3 (5%)	3 (5%)	6 (5%)
**Fight finish**							
Decision	13 (48%)	19 (51%)	32 (50%)		55 (86%)	50 (88%)	105 (87%)
TKO	7 (26%)	9 (24%)	16 (25%)		9 (14%)	5 (8.8%)	14 (12%)
Submission	4 (15%)	7 (19%)	11 (17%)		0 (0%)	0 (0%)	0 (0%)
KO	3 (11%)	2 (5%)	5 (8%)		0 (0%)	2 (4%)	2 (2%)
**Fight notice**							
6–8 weeks	8 (30%)	18 (49%)	26 (41%)		33 (52%)	29 (51%)	62 (51%)
3–5 weeks	10 (37%)	8 (22%)	18 (28%)		16 (25%)	12 (21%)	28 (23%)
>8 weeks	7 (26%)	10 (27%)	17 (27%)		13 (20%)	13 (23%)	26 (21%)
0–2 weeks	2 (7%)	1 (3%)	3 (5%)		2 (3%)	3 (5%)	5 (4%)

MMA = mixed martial arts, MT = Muay Thai, KO = knockout, TKO = technical knockout.

**Table 2:** Body mass and rapid weight change data for mixed martial arts and Muay Thai by contest winners and losers. Data are presented as medians (interquartile ranges) and means ± standard deviations. Effect sizes are provided using Hedges’ g for mean comparisons and rank biserial for median comparisons.

	MMA					MT			
Variable	Winners	Losers	Overall	Effect Size [95% CIs]/ *p* Value		Winners	Losers	Overall	Effect Size [95% CIs]/ *p* Value
	n = 27	n = 37	n = 64			n = 64	n = 57	n = 121	
**Body mass,** **–7 days (kg)**	74	75	74	−0.04 [−0.32, 0.24]/ 0.8		68	68	68	0.10 [−0.11, 0.30]/ 0.3
	(67, 78)	(67, 80)	(66, 79)			(63, 78)	(60, 76)	(63, 77)	
**Body mass,** **–24 h (kg)**	71	73	72	−0.06 [−0.34, 0.22]/ 0.7		67	66	67	0.07 [−0.13, 0.27]/ 0.5
	(65, 77)	(67, 78)	(66, 78)			(62, 75)	(58, 73)	(61, 74)	
**Official** **weigh-in (kg)**	67	70	69	−0.06 [−0.33, 0.23]/ 0.7		64	64	64	0.06 [−0.14, 0.26]/ 0.6
	(63, 74)	(65, 76)	(64, 76)			(61, 72)	(58, 72)	(59, 72)	
**Secondary** **weigh-in (kg)**	73	76	73	−0.07 [−0.34, 0.22]/ 0.7		69	68	69	0.12 [−0.09, 0.32]/ 0.3
	(66, 78)	(69, 80)	(68, 79)			(65, 79)	(61, 75)	(62, 76)	
**Missing**	**1**	**0**	**1**			**1**	**1**	**2**	
**RWL,** **–24 h (kg)**	2.6	2.0	2.4	0.13 [−0.16, 0.40]/ 0.4		1.8	1.5	1.7	0.15 [−0.05, 0.35]/ 0.15
	(1.8, 3.3)	(1.4, 3.2)	(1.4, 3.2)			(1.2, 2.4)	(0.7, 2.4)	(1.0, 2.4)	
**RWL,** **–7 days (kg)**	4.8	4.1	4.5	0.21 [−0.07, 0.47]/ 0.2		3.7	3.1	3.4	**0.27 [0.07, 0.45]/** 0.01 *
	(3.8, 6.2)	(2.7, 5.4)	(3.1, 6.0)			(2.8, 5.0)	(1.6, 4.5)	(2.3, 4.5)	
**RWG (kg)**	4.6 ± 2.0	4.2 ± 2.4	4.4 ± 2.3	0.19 [−0.31, 0.68]/ 0.5		4.1 ± 1.9	3.0 ± 1.9	3.6 ± 2.0	**0.57 [0.21, 0.94]/** 0.002 *
**Missing**	**1**	**0**	**1**			**1**	**1**	**2**	
**RWG/RWL (%)**	0.94	0.91	0.93	0.01 [−0.28, 0.29]/ >0.9		1.08	0.86	1	**0.22 [0.02, 0.41]/** 0.038 *
	(0.77, 1.05)	(0.73, 1.22)	(0.75, 1.14)			(0.86, 1.32)	(0.60, 1.19)	(0.73, 1.24)	
**Missing**	1	1	2			1	4	5	

MMA = mixed martial arts, MT = Muay Thai, RWL = rapid weight loss, RWG = rapid weight gain, CIs = confidence intervals. * Difference between contest winners and losers, *p* < 0.05 *. Missing = observations that were missing from the dataset or could not be calculated for the variable in the row above.

**Table 3:** Mixed logistic regression analyses were conducted, controlling for age, to investigate the relationship between rapid weight change variables in males and females and the likelihood of winning. An OR of <1 indicates a greater likelihood of losing, while an OR > 1 indicates a greater likelihood of winning. A total of 263 responses were collected, comprising 204 males (winners = 106, losers = 98) and 59 females (winners = 36, losers = 23).

	Model 1		Model 2		Model 3		Model 4	
Predictors	ORs (95% CIs)	*p*	ORs (95% CIs)	*p*	ORs (95% CIs)	*p*	ORs (95% CIs)	*p*
Age	0.92 (0.87–0.97)	0.004 *	0.92 (0.87–0.97)	0.002 *	0.91 (0.85–0.96)	0.002 *	0.91 (0.86–0.97)	0.002 *
RWL–7 days (%): Female	1.28 (1.09–1.51)	0.003 *						
RWL–7 days (%): Male	1.05 (0.96–1.15)	0.278						
RWL–24 h (%): Female			1.57 (1.19–2.07)	0.001 *				
RWL–24 h (%): Male			1.09 (0.95–1.26)	0.219				
RWG (%): Female					1.31 (1.11–1.54)	0.001 *		
RWG (%): Male					1.09 (0.99–1.20)	0.072		
RWG/RWL ratio: Female							1.01 (1.00–1.02)	0.033 *
RWG/RWL ratio: Male							1.00 (1.00–1.01)	0.427
**Random Effects**								
Athletes (n)	185		185		183		179	
Total responses (n)	263		263		261		253	
Marginal R^2^/ Conditional R^2^	0.10/0.20		0.11/0.19		0.11/0.22		0.07/0.17	

RWL = rapid weight loss, RWG = rapid weight gain, CIs = confidence intervals, ORs = odds ratios, * Significant effect on contest outcome, *p* < 0.05 *.

## Data Availability

The data are not publicly available due to privacy and ethical restrictions.

## Supplementary Materials

The following supporting information can be downloaded at: https://www.mdpi.com/article/10.3390/sports12100280/s1, Supplementary File S1: Weight Management Questionnaire; Supplementary File S2: Mixed Effects Logistic Regression Diagnostics for Models 1–4.
