# Supplementary material for: Rapid Weight Changes and Competitive Outcomes in Muay Thai and Mixed Martial Arts: A 14-Month Study of 24 Combat Sports Events
Source: Sports (Basel). 2024 Oct 16;12(10):280. doi: 10.3390/sports12100280 (PMC11511017; doi:10.3390/sports12100280)
Supplement: Supplementary file 1 [file sports-12-00280-s001.zip › Supplementary File S1.pdf]

WEIGHT MANAGEMENT QUESTIONNAIRE

What is your full name?

____

In what category do you compete?

- ○ Mens
- ○ Womens

What sport will/did you compete in?

- ○ Boxing
- ○ Mixed martial arts
- ○ Muay Thai

How many week's notice did you receive for this fight?

- ○ 0-2 weeks
- ○ 3-5 weeks
- ○ 6-8 weeks
- ○ >8 weeks

What was your official weigh-in weight in kilograms (i.e 70.4kg)?

____

What was your body weight in kilograms **7 days** before weigh-ins (i.e 78.3kg)?

____

What was your body weight in kilograms **the day before** weigh-ins (i.e 73.4kg)?

____
