# Supplementary material for: Rapid Weight Changes and Competitive Outcomes in Muay Thai and Mixed Martial Arts: A 14-Month Study of 24 Combat Sports Events
Source: Sports (Basel). 2024 Oct 16;12(10):280. doi: 10.3390/sports12100280 (PMC11511017; doi:10.3390/sports12100280)

Mixed Effects Logistic Regression Diagnostics

Model 1

Rapid weight loss -7 days (%)

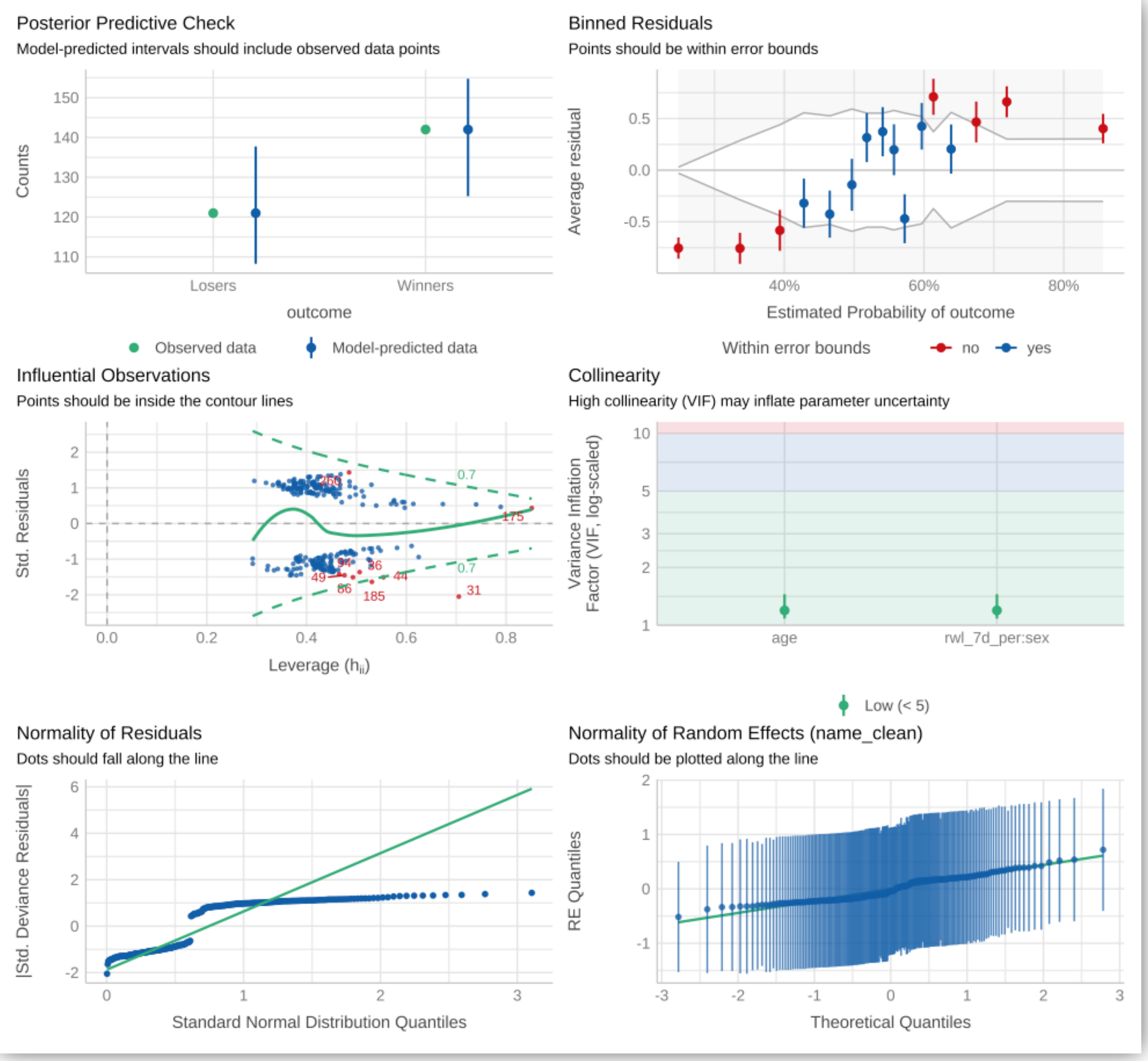

Model 2

Rapid weight loss -24 hours (%)

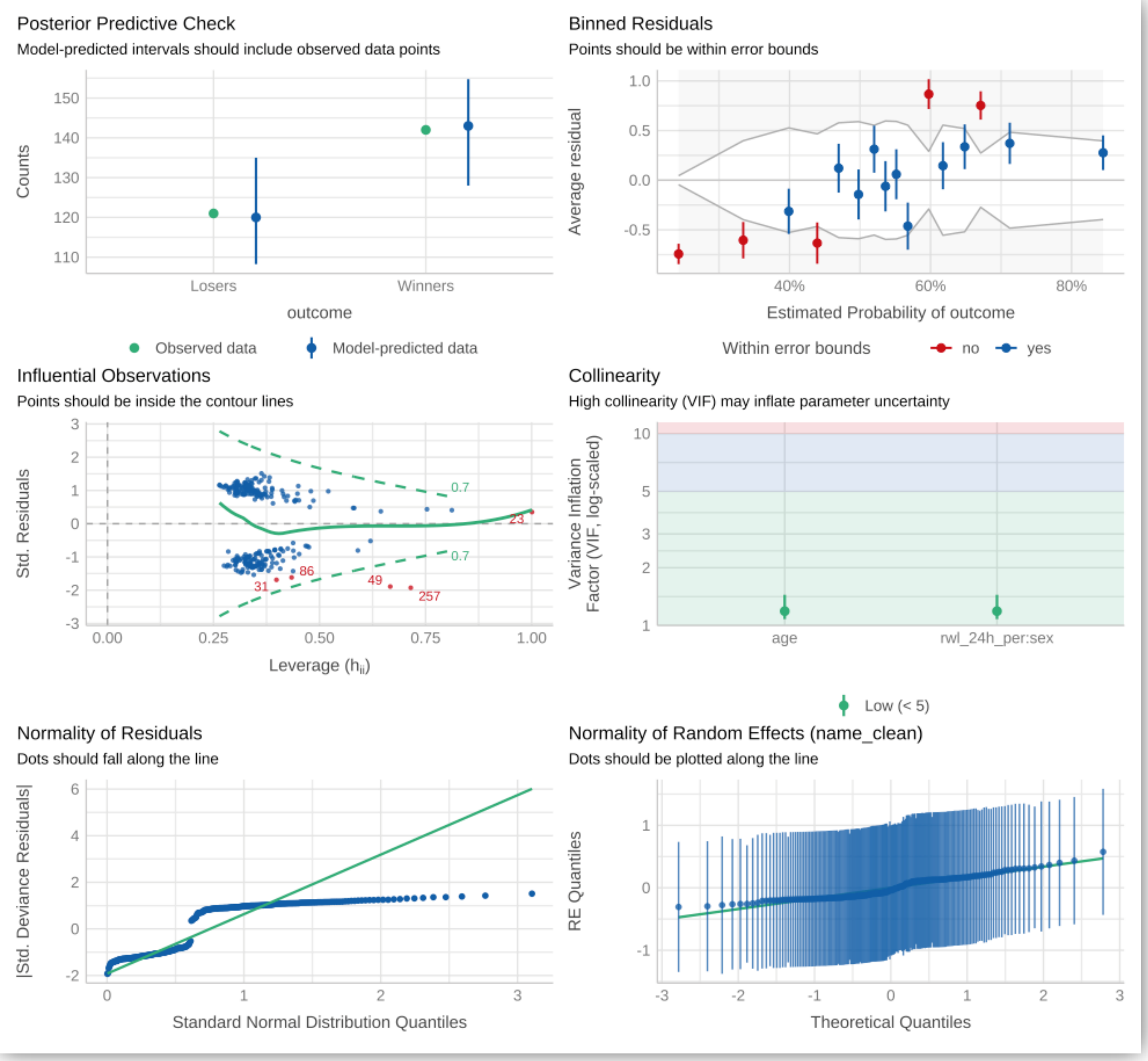

Model 3

Rapid weight gain (%)

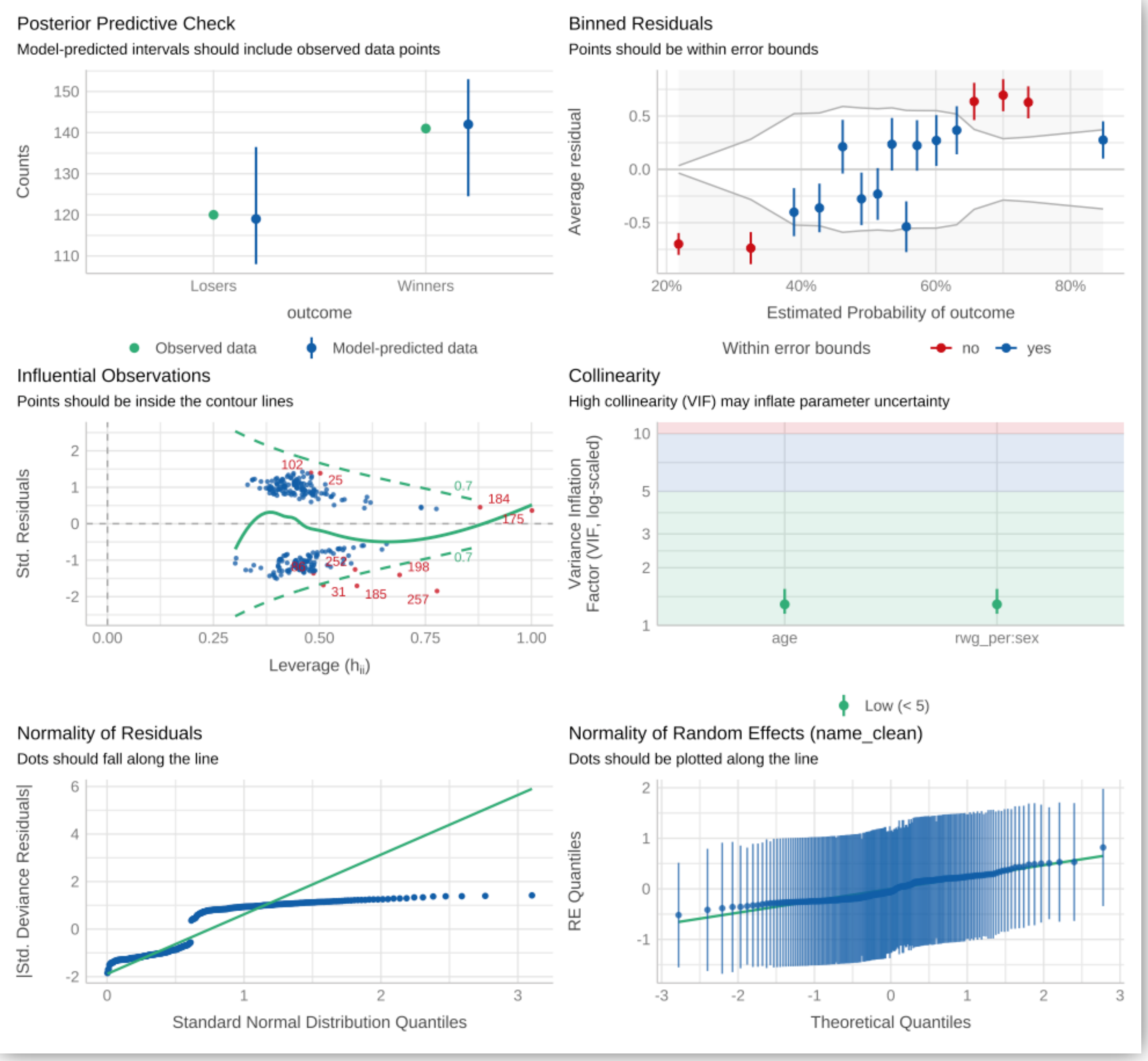

Model 4

Rapid weight gain/ rapid weight loss -7 days ratio

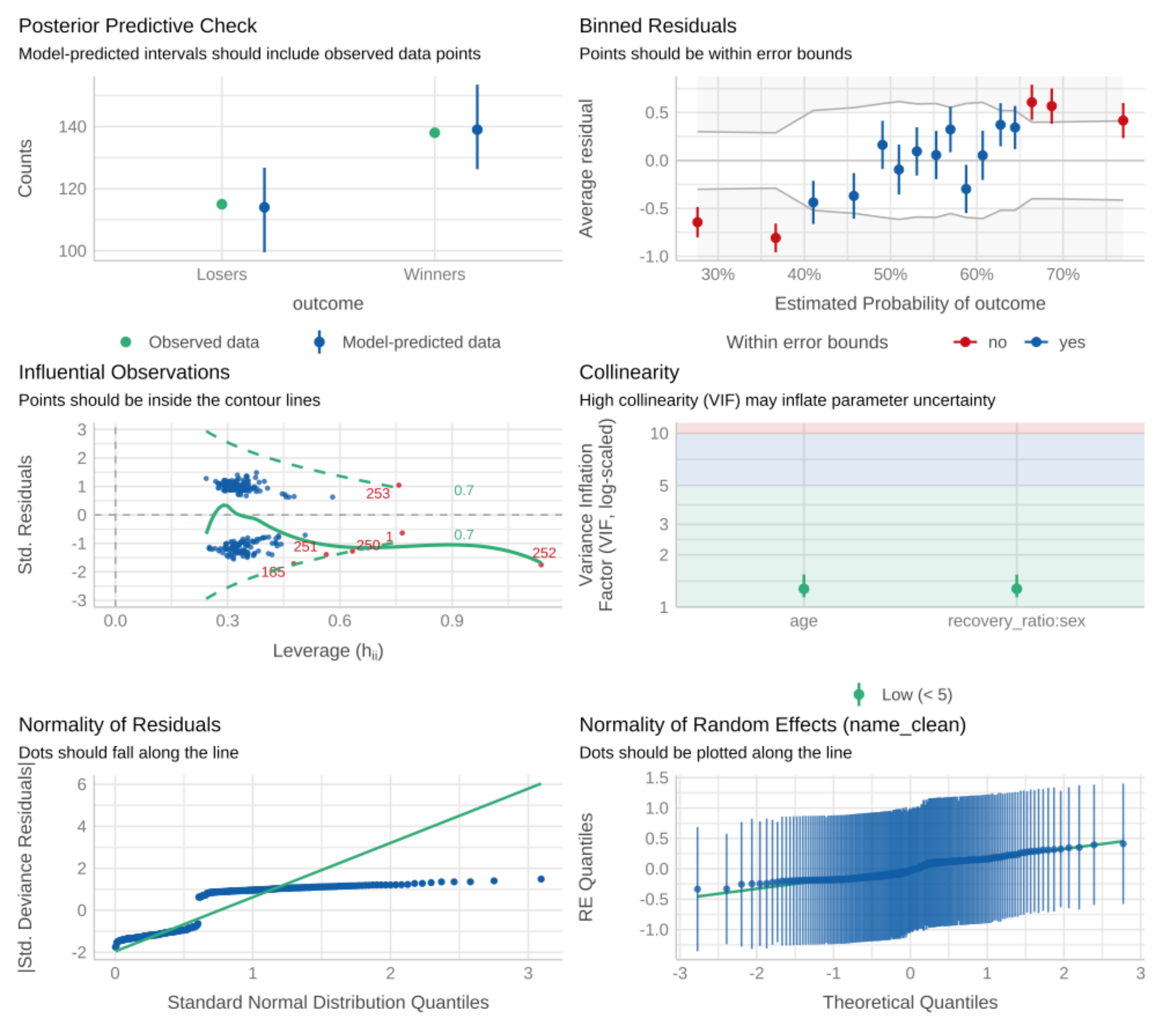

Supplement: Supplementary file 1 [file sports-12-00280-s001.zip › Supplementary File S2.pdf]
